# Radiation injury is a potentially serious complication to fluoroscopically-guided complex interventions

**DOI:** 10.2349/biij.3.2.e22

**Published:** 2007-04-01

**Authors:** LK Wagner

**Affiliations:** Department of Diagnostic and Interventional Imaging, The University of Texas Medical School at Houston, Houston, Texas, USA.

## Abstract

Radiation-induced injury to skin is an infrequent but potentially serious complication to complex fluoroscopically-guided interventional procedures. Due to a lack of experience with such injuries, the medical community has found fluoroscopically-induced injuries difficult to diagnose. Injuries have occurred globally in many countries. Serious injuries most frequently occur on the back but have also occurred on the neck, buttocks and anterior of the chest. Severities of injuries range from skin rashes and epilation to necrosis of the skin and its underlying structures. This article reviews the characteristics of these injuries and some actions that can be taken to reduce their likelihood or seriousness.

## INTRODUCTION

A 154 kg patient presented at the Emergency Center complaining of a prolonged rash located medially on the upper part of his back. The rash was almost rectangular and well demarcated, measuring about 40 mm by 60 mm. The affected skin had a central blackened area about 10 mm in dimension. The rash first appeared about six months previously; initially it was red and very itchy. The patient sought medical help shortly afterwards. The dermatitis of unknown aetiology was treated topically. With time, the rash worsened. Further medical assistance was sought but was ineffective. The patient admitted himself to an emergency centre. That visit also proved unsatisfactory in diagnosing the cause of the injury. Now, the patient presented at a different emergency centre. The patient had a history of heart disease and about one month prior to the onset of the rash had undergone a complex coronary angioplasty and stent procedure. By the recollection of the patient’s spouse, the procedure lasted about six hours. The equipment used for the procedure was a state-of-the-art flat-panel digital angiography system. The patient had never been advised that radiation received from that prolonged study could cause such an injury. Therefore, with the rash developing several weeks later he had no reason to suspect that the treatment for his heart condition might have any significance. It was a classic case of radiation injury from fluoroscopically-guided coronary intervention. Many such cases with similar scenarios have occurred in the past decade [1-5]. The cases frequently involved delayed diagnosis of a well-demarcated rash, with a prolonged and intractable progression to a necrotic wound. Even so, diagnosis of the lesion’s aetiology has proven difficult. In some situations, after a prolonged period without diagnosis, a member of the patient’s family performed the research necessary to discover the cause.

**Figure 1:**
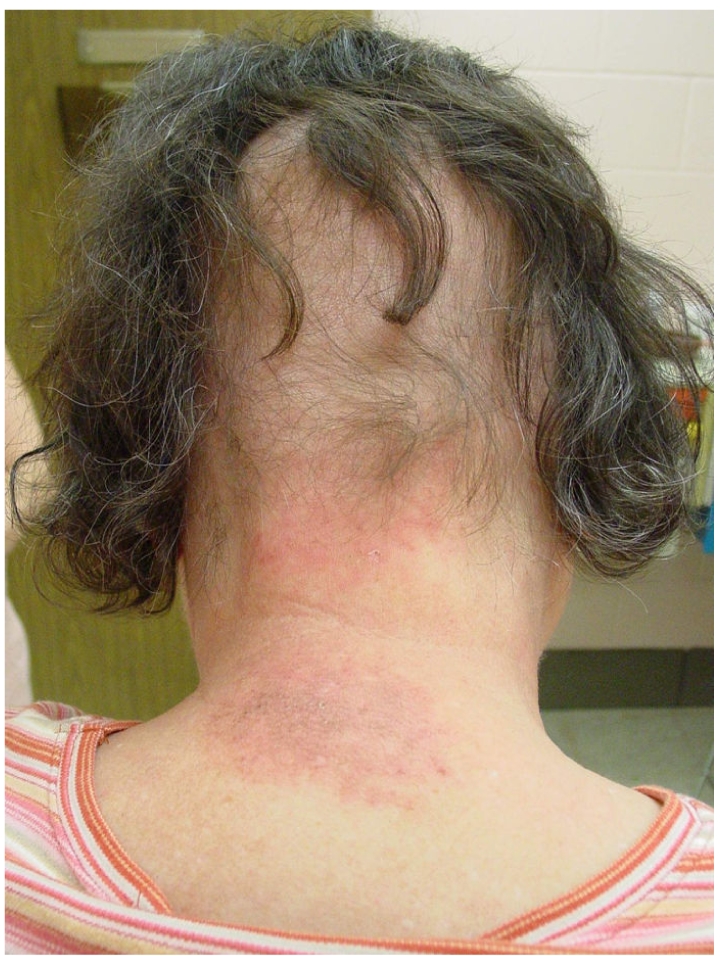
Injury on neck from neurointervention (Reproduced with permission from anonymous donor).

**Figure 2:**
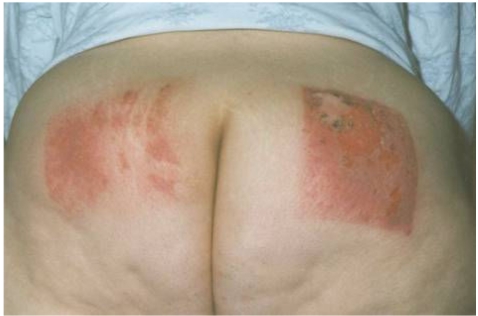
Radiation injuries from bi-plane uterine embolisation procedure (Photo courtesy of Thomas B. Shope, United States Food and Drug Administration).

**Figure 3:**
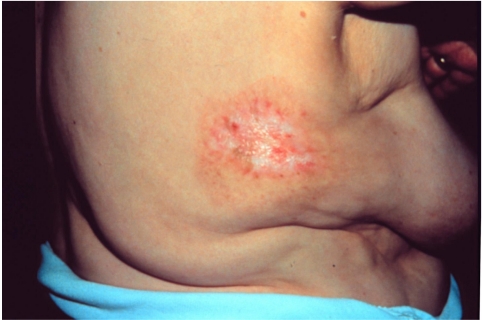
Injury to right side of patient at 11 months after percutaneous transluminal coronary angioplasty (Reproduced with permission from Koenig *et al *[1]).

**Figure 4:**
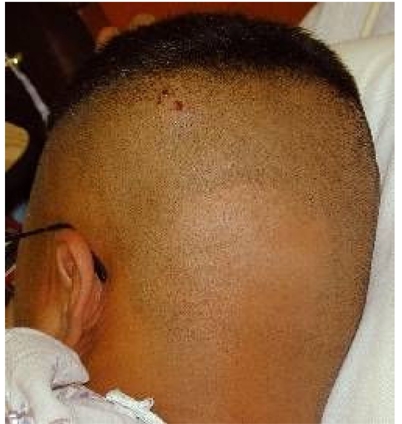
Epilation following embolisation of a dural AV-fistula. Affected area is circular area of hair loss in shaved area of head (head shaved for gamma knife procedure). (Reproduced with permission from Koenig TR, Wagner LK, Mettler FA, Wolff D. Radiation Injury to the Skin Caused by Fluoroscopic Procedures: Lessons on Radiation Management, Scientific Exhibit, Annual Meeting of the Radiological Society of North America, 2000).

Hundreds of injuries from complex fluoroscopically-guided interventions have been reported, ranging in severities from mild erythema and hair loss to deep skin necrosis, sometimes involving deeper tissues to the level of bone and spine. Severe injuries have occurred worldwide from Europe to the Americas, and Asia [1-12]. The equipment involved has ranged from poorly designed systems to contemporary state-of-the-art machines. Severe injuries have occurred, ranging from the neck to the buttocks (Figures 1-2). Injuries have occurred anteriorly [12] and on the sides of the torso (Figure 3), but most have occurred posteriorly due to the conventional orientation of the fluoroscope. Conspicuously, the author knows of no severe injuries in the scalp, although depilation has been observed on many occasions (Figure 4).

**Figure 5:**
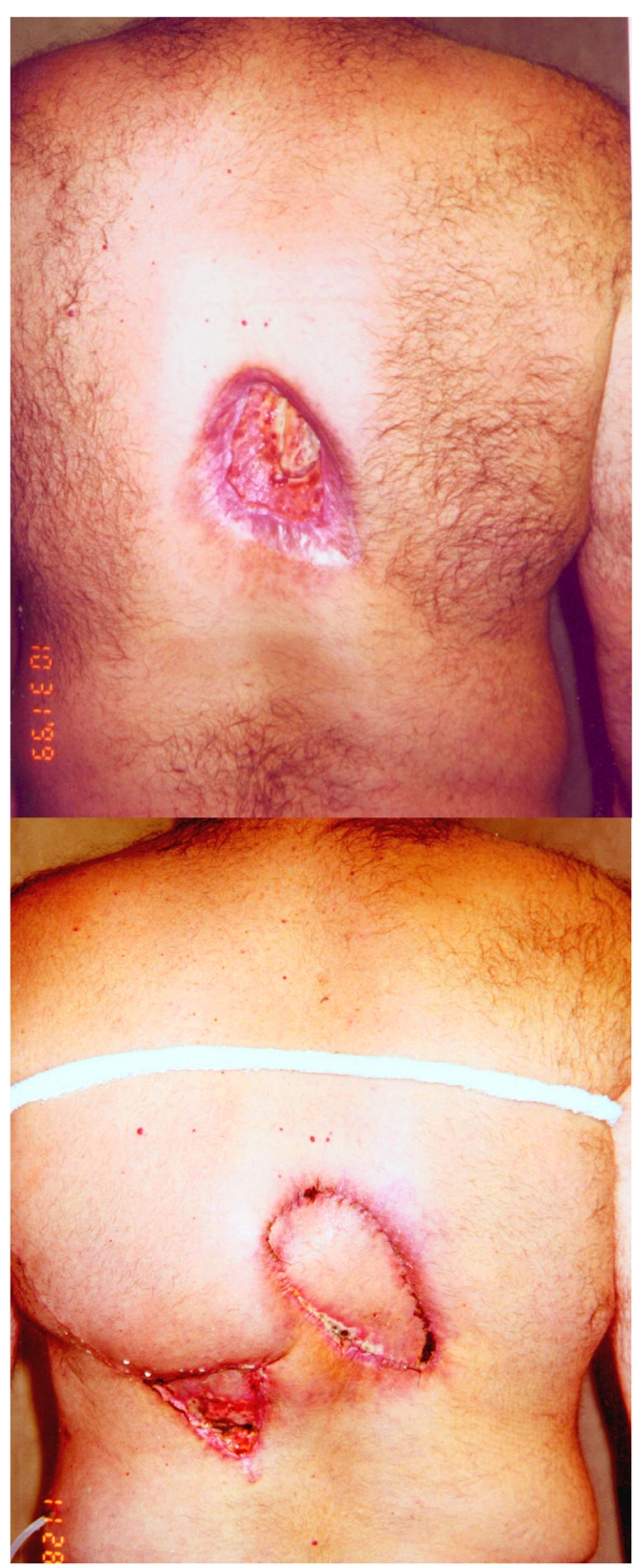
Injury following three procedures involving transjugular intrahepatic portosystemic shunt placement, demonstrating disfigurement after surgical correction. (Reproduced with permission from Koenig *et al* [1]).

The pain and suffering associated with severe injuries and their inevitably prolonged wound management is only one element in the scale of effects. The medical treatment sometimes involves surgical grafting that results in permanent disfigurement and compromised mobility (Figure 5). In some cases the family’s lifestyle is radically altered. This includes daily changes of wound dressings, limited ability to perform simple tasks, inability to work, loss of income and indebtedness due to high medical costs and loss of employment. In some cases, the patient must learn to sleep in awkward positions because the wound prevents the patient from reclining in a normal way. Psychological depression in both the patient and the patient’s closest family members is a further complication that has sometimes required treatment. In some cases, the pain associated with the injury is permanent and the patient requires a lifetime of medication and treatment for pain.

The medical benefits of complex fluoroscopically-guided interventions are well established. They include lower morbidity with reduced risk of mortality with a much shortened recovery period when compared to that of conventional surgical methods. It is estimated that about two million such procedures are performed worldwide each year. Since only hundreds of injuries are known, the occurrence of radiation injury as a complication to these procedures is extremely rare. Consequently, the concern about radiation and the motivation for improved techniques to avoid such complications is not in the frequency of the occurrence; rather it is the severity of the complication that warrants improved dose-limiting techniques. An added impetus for better radiation management is to prevent an increase in the occurrence of these injuries as procedures become more aggressive and complex with future advances. Concern over radiation injury should not become a cause for a physician to prematurely terminate a procedure that is deemed necessary to save the life of a patient.

On the other hand, using equipment that is appropriately designed for complex interventions and assuring that medical personnel are properly trained in the use of that equipment as well as in methods on how to limit dose during such procedures is a reasonable medical goal. This will ensure that the risks of radiation are appropriately minimised. The benefits will be the avoidance of injury in many cases and the reduced potential for long-term neoplastic effects of radiation. An added benefit is limited radiation exposure to personnel resulting in a lower carcinogenic risk for them.

## THE CHARACTERISTICS OF RADIATION INJURY FROM FLUOROSCOPY

Although a radiation injury is often referred to as a “burn”, the development of the injury is considerably different from that of a thermal or chemical burn [13-14]. Since the physical appearance of the injury reminds one of a thermal “burn”, it is natural to think about the causes of the wound in the same context. Thus, it is natural to try to identify some thermal or chemical agent with which the patient has recently come into contact. As the wound is often sharply demarcated, one naturally looks for agents that would cause sharp borders. This often leads to frustration and misdiagnoses. The situation is often exacerbated by attempts to treat the wound in the same manner as a thermal or chemical burn. For thermal or chemical injuries, the wound develops rapidly once the agent of cause is removed. Within a matter of days, the full extent of development is usually known. Results of treatment begin to appear in a short interval and progress relatively rapidly, over a period of days, sometimes weeks. Radiation injuries, especially those involving severe injuries, do not have these characteristics.

Most frequently, in the case of fluoroscopic radiation skin injury, symptoms of the injury are not promptly apparent. This is because damage to the cells by ionising radiation is very different from that caused by heat and chemical agents.

Heat and chemical agents cause a global damage that affect the entire cell and groups of cells by introducing energy. This causes molecules to break apart. Chemical and biochemical reactions ensue. Heat and chemicals must progress through all superficial layers of cells to penetrate to deeper layers. Thus with heat and chemicals, every cell in the superficial structures of the contact zone of the skin is wholly and adversely affected. Nerves sense this and immediately signal the individual to reduce contact with the offending agent. Fluoroscopic X-ray radiation does not do that.

An X-ray beam is comprised of billions of individual X-ray photons. An X-ray photon can be considered to be an uncharged particle of pure energy with no mass. It is about the size of an atom. These properties allow the photon to bypass many layers of cells without interacting in the cells. When a photon does happen to interact in a cell, it interacts with electrons in individual atoms or molecules inside the cell. Thus, the cell can be injured internally in a very localised area without damage to its outer structures. In this way, the structure of the cell often remains intact but the replicating capacity of the cell can be compromised. This, in fact, is a characteristic of cell damage by ionising radiation. In general, immature cells that reproduce frequently are more susceptible to the lethal effects of radiation than mature cells.

As a result of the internal cellular damage affecting replication, patients who undergo very high-dose interventional procedures often have no sense of a radiation skin injury before they leave the hospital. However, the basal cell layer of the epidermis might have damages that will compromise skin renewal. As the skin proceeds through its normal replication and renewal process, it will find itself incapable of completing this function. As the process takes many days to develop, there will be a characteristic delay between the induction of the injury and the recognition of symptoms, which begin as a rash. The delay is typically, but not always, about two to three weeks before symptoms emerge and three to four weeks before it is sufficiently irritating for the patient to see a doctor. Thus, physicians and patients do not usually associate the rash with the angiographic procedure.

In a few cases, symptoms of fluoroscopically-induced radiation injury have occurred promptly, within a matter of hours. Reported symptoms are pain on the back or a rash. The prompt rash is thought to be caused by a mechanism different than that described previously. In short, the ionisation caused by the radiation is thought to lead to an activation of histamine-like substances, resulting in a dilation of capillaries [14]. This type of rash often fades after a day or so. However, depending on the amount of radiation delivered, the rash may seem to blend with that of the delayed erythema associated with damage to the basal cells of the epidermis. While early symptoms have been reported, they either occur infrequently or they are not usually recognised.

Skin erythema is one of the first symptoms to be noticed because the affected cells are superficial and are in a state of continual replication. However, even if a lethal amount of radiation is delivered to a cell, the cell might still continue to function for a time. Eventually, however, the cell dies and must be replaced. This process occurs on a different time scale for different cells. For instance, the epithelial cells of the vascular structures of the dermis might be damaged. As time evolves, these cells need to be replaced. However, the repair mechanisms might be compromised and this results in a shutdown, rather than a replenishment of the blood supply to the skin. Edema that slowly develops might also contribute to vascular collapse. The ultimate result is necrosis that begins to be evident within months after the angiographic procedure, with the time course dependent on many factors like radiation dose and skin type.

Table 1 provides a summary of some observed patterns of radiation damage to the skin. With the exception of skin cancer, the important lesson is that these effects do not occur unless the dose of radiation is greater than the minimum necessary to cause sufficient damage. Also, because the temporal course of radiation injury by fluoroscopy is delayed and is quite unlike that for thermal injury, it is possible to reach a diagnosis by analysing the relationship of the temporal progression from the time of the previous fluoroscopic procedure. This coupled with the shape and location of the injury leads to a reliable diagnosis. The injury must be located in the area of the skin where the radiation enters the patient and the shape of the injury will depend on how the radiation was delivered. If the beam was stationary, never adjusted for collimation and located over the same area of skin for most of the procedure, then the injury will take on the shape of the X-ray port and will have sharply demarcated borders. The shape might be rectangular or circular, depending on the type of collimator. Deviations from this, e.g., re-oriented beam or adjusted collimators, may result in less sharply demarcated borders or more oddly shaped injuries (e.g., Figure 1 versus Figure 2). Such an analysis is likely to be sufficient for diagnosis. This will both avoid the need for biopsy and the associated complications of an open wound in skin already damaged by radiation.

**Table 1:** Potential effects in skin from fluoroscopy (adapted from Wagner *et al* [15] and revised according to information provided in private communication with Hopewell JW, 1999).

**Effect**	**Single-dose threshold (Gy)**	**Onset**
Early transient erythema	2	˜2 – 24 h
Main erythema	6	˜10 d
Temporary epilation	3	˜3 wk
Permanent epilation	7	˜3 wk
Dry desquamation	14	˜4 wk
Moist Desquamation	18	˜4 wk
Secondary Ulceration	24	>6 wk
Late erythema	15	8 -10 wk
Ischemic dermal Necrosis	18	>10 wk
Dermal atrophy (1st phase)	10	>12 wk
Dermal atrophy (2nd phase)	10	>1y
Induration (invasive fibrosis)	10	
Telangiectasia	10	>1y
Dermal necrosis (late phase)	>12?	>1y
Skin cancer	None known	>5y

## HOW TO MINIMIZE RISK FOR RADIATION-INDUCED INJURY IN PATIENTS

Radiation management for the patient has three phases: before the procedure begins, during the procedure and after the procedure is over.

### Before the procedure

Important considerations before a procedure are:

1. the skill sets of the physician
2. the physicians’ and the technologists’ knowledge about their angiographic machine
3. the medical history of the patient
4. the likely difficulty of the procedure
5. the body habitus of the patient.

Some injuries have been associated with procedures for which the physician was inexperienced and not sufficiently trained. Insufficient experience leads to prolonged use of radiation. Conversely, well-trained and experienced physicians are likely to be more efficient in completing the procedure. Training and experience in the technical aspects of a medical intervention are important components of radiation management. Physicians must be properly trained and experienced in procedures before attempting them and must exercise prudent judgment when attempting procedures that challenge their skill sets. They should seek assistance early in a procedure if the difficulty presents a new or unexpected challenge.

Training includes lessons in the prudent use of fluoroscopy and fluorography. Learning to limit fluoroscopy to the minimum time necessary for every engagement of the switch is essential. Prudently limiting serial runs in number and in duration is also essential.

Setting up the machine for a procedure requires not only knowledge about radiation management, but also training on how to set up a particular machine to make use of that knowledge. Knowing the options and capabilities of a particular machine is essential. Many features can be adjusted during the procedure to reduce radiation use or to improve image quality, depending on the demands of the situation.

Some patients are at greater risk for radiation injury than others. Some drugs, such as actinomycin D and Adriamycin^®^, are known to increase sensitivity to X rays [1, 16]. Some rare health conditions render patients highly sensitive to radiation, e.g., patients with the homozygous form of the ataxia telangiectasia gene [1,16]. Diseases such as collagen vascular diseases and diabetes mellitus are suspected in rendering patients more susceptible to injury [1, 17, 18]. Diabetes compromises the vascular supply and this leads to a greater risk for long-term complications. The reasons why some patients with collagen vascular disease are more sensitive to radiation are unknown. Medications that the patient is taking may be one reason for the heightened sensitivity [9]; but the sensitivity might also be related to the status of the disease at the time of the procedure. However, having the disease does not absolutely predispose patients to heightened sensitivity. Only a few patients with collagen vascular disease have been identified to have greater radiation sensitivity [1, 9, 17].

If the patient has had previous fluoroscopically-guided procedures, it is wise to examine his or her skin for erythema or residual radiation injury from those procedures. A previous injury may never have been reported by the patient as it might not have caused sufficiently severe symptoms. It may have healed into a slightly scarred or discoloured area and might not be in an area where the patient can see it. If a residual injury is identified, that skin area will be at heightened risk for injury. This should be brought to the attention of the patient. Furthermore, the physician might be able to plan the current procedure to avoid irradiation of that skin area.

If the procedure is likely to be difficult, requiring a prolonged course of fluoroscopy with more than the usual number of imaging run-offs, then the patient will be at risk for an unusually high radiation dose to the skin. This is especially true if the patient is large. To compensate for the increased absorption of radiation by the increased body mass, the X-ray machine will automatically adjust the radiation output to high levels. Thus, radiation dose will accumulate much faster when the X-ray beam must traverse increased body mass. This occurs not only in large patients, but also in smaller patients for whom the beam angle is tilted in oblique, cranial-caudal or caudal-cranial orientations.

When the patient is at risk for a high dose procedure, obtaining informed consent should be considered. Some suggestions and considerations for the informed consent are provided in Table 2

**Table 2:** Potential radiation effects to consider in informed consent.

Hair loss Usually temporary; regrowth of hair may be incomplete.
Skin rashes Infrequent, on very rare occasions may result in tissue breakdown and possibly severe ulcers or wounds that require surgical intervention.
Slightly elevated risk for cancer Occurs later in life. This risk is typically low compared to the normal incidence of human cancer.
Cataracts occur rarely and are a risk only for neurointerventional procedures.

### During the procedure

A friend once told me that for angiographic procedures, radiation should be managed in the same context as iodinated contrast agents [Stephen Balter, 2005]. All angiographers can relate to the risks associated with iodine. The amount of iodine administered to a patient is monitored and the physician makes a benefit/risk decision regarding the amount to be used. The physician also knows how to use iodine wisely, so as to avoid situations that might place the patient at unnecessary risk. Radiation is similar: the amount delivered should be monitored and the physician must know how to use it wisely so as not to place the patient at unnecessary risk.

Wagner and Archer have reviewed methods of radiation management [19] and these methods have been reviewed in many other articles [2-5, 20]. This paper will highlight important lessons of radiation management as they relate to observed injuries. For a more thorough discussion, the reader is referred to the referenced publications.

#### Thick tissue masses

Injuries are often associated with large patients and beam projections through thick body masses, as is evident for many injuries shown in this review. This occurs when patients are large, beam angles are steep, or arms or other obstructing body parts are in the path of the beam. The entrance dose rate increases for both fluoroscopy and fluorography (serial imaging such as runoffs or cine). The cause of the increased radiation rate is two-fold. First, the goal of all fluoroscopy and fluorography is to produce a residual radiation beam on the exit-beam side of the patient sufficient to result in a satisfactory image for the task. However, X rays do not readily penetrate through patients. Typically for abdomens, less than 1% of the radiation that enters a patient actually penetrates through to make the image. The rest of the radiation interacts inside the patient. Fluoroscopic X-ray energy absorption is greatest at the surface where the beam enters the patient, about 100 times greater than at the exit surface when the projection is through a typical abdomen or mediastinum. For thicker body masses, more radiation has to be delivered in order to get the same amount through. Typically for every 3-5 cm of tissue that has to be traversed, the radiation output must increase by another factor of two. By governmental regulation, the output of fluoroscopy is usually capped at a limited maximum output. However, typically there is no such cap or limit placed on serial runs. So, for thick body masses the fluoroscopy output might be operating at the maximum allowed level while the serial run output is not limited and could be running at dangerously high levels, as has occurred in some cases of injury.

**Figure 8:**
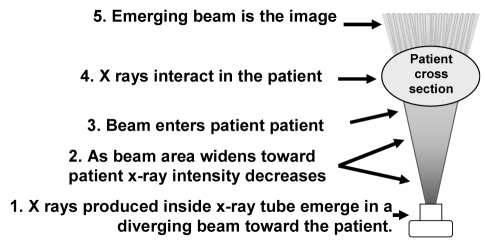
The X-ray beam. X rays are produced in a small area inside the X-ray tube. They emerge in a diverging beam. The beam is most intense at positions closest to the source. (Adapted with permission from Wagner *et al* [19]).

The second reason why dose rates on the skin are higher is due to the proximity of the entrance skin surface to the X-ray source. X rays emanate from a tiny point inside the X-ray tube. The beam diverges from this point and expands into an ever widening area as distance from the source increases (Figure 8). As the skin gets closer to the source, the area of the beam is smaller. This means that all the X rays are confined to a smaller area as the source is approached, resulting in an increasing intensity of radiation. Big patients, thick body masses and arms, all contribute to situations where the skin surface of the patient is closer to the source than for thin body sections.

To help abate large dose build-up under the situations described above, the following principles can be applied:

1. Assure that the patient’s skin surface is maintained at a reasonable distance from the source.
2. Rotate the beam to a different angle so as not to irradiate the same skin site for a prolonged period of time.
3. Position patients so that the arms can be moved out of the X-ray field.
4. Try not to use beam angles where the female breast is directly exposed to the entrance beam.

**Figure 6:**
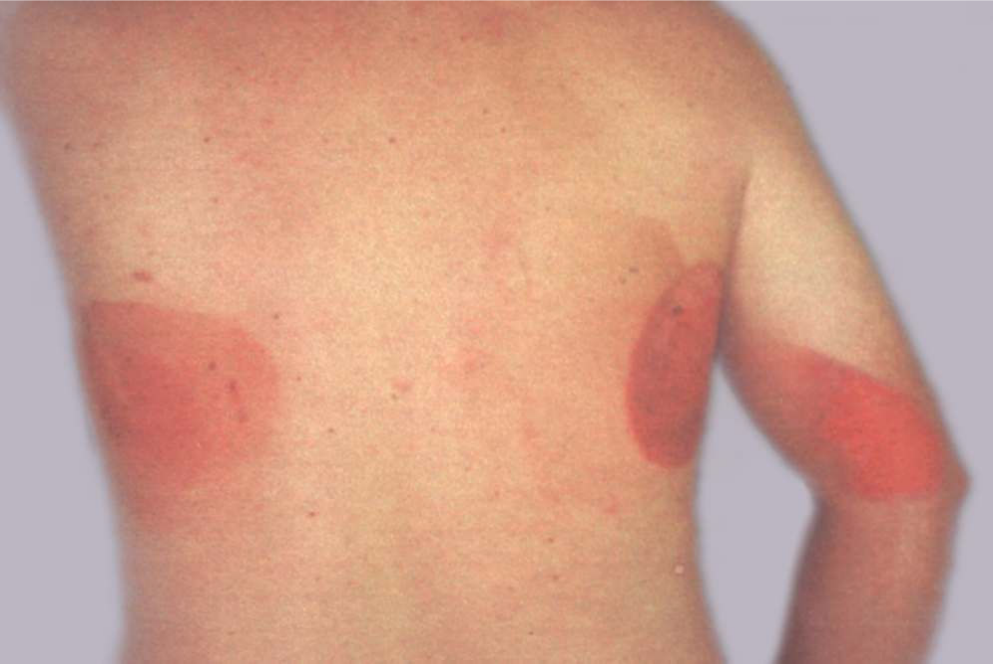
Injuries to back and arm from multiple prolonged electrophysiological and ablation procedures with bi-plane fluoroscopy. Wounds on back healed into scarred areas while injury on arm required grafting. (Reproduced with permission from Vlietstra *et al* [4]).

Execution of these principles requires prudent judgment. The relationship of the source to the patient has boundary conditions that are imposed by the situation. If an isocentric configuration is used in a cardiac procedure, the heart of the patient will be at a fixed position relative to the source, which in turn determines the position of skin surface in relation to the source. But if an isocentric configuration is not required, the table of the patient might be raised somewhat. The table height will depend on the height of the physician who must maintain a comfortable working level. Rotating the beam is often possible, but in some cases this will reduce the visibility of the lesion and might compromise the quality of the procedure. Arms can usually be moved away from the path of the beam and efforts to do so with arm boards or other methods are highly recommended. Several cases of arm injuries (Figures 6, 9, 10) have been reported [1, 4, 8]. Staff should be trained to look for arms in the field so that they can alert the physician of the circumstance and correct it as necessary. Breast cancer from high doses delivered to the mammary tissues of female patients is a known risk [21]. Young women or girls are at greatest risk [22]. Figure 11 shows an injury to the flank of a 17-year-old girl from an electrophysiological and ablation procedure. The skin dose was obviously very high. Due to the close proximity of the right breast, dose to that breast was also very high from both direct irradiation and indirect scattered radiation. Avoiding exposure to the breast, especially direct entrance beam exposure, is highly recommended. Collimating to the area of interest is an effective way to reduce scattered radiation.

**Figure 7:**
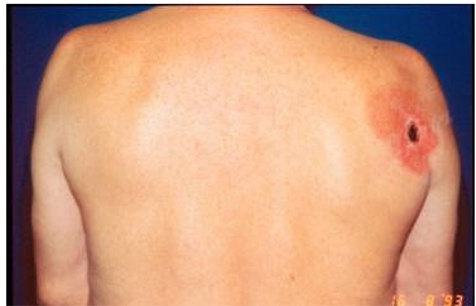
Injury to shoulder from percutaneous transluminal coronary angioplasty. (Reproduced with permission from Koenig *et al* [2]).

**Figure 9:**
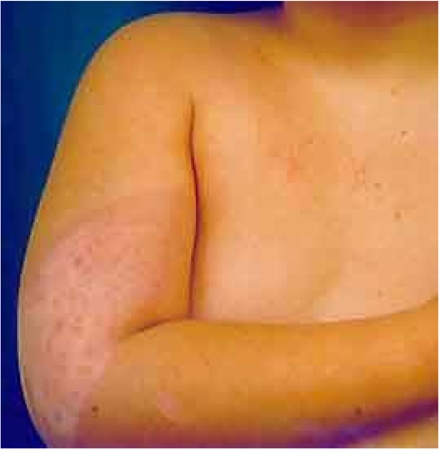
Arm of 7-year-old girl after cardiological ablation procedure. Injury to arm occurred due to added attenuation of beam by presence of arm and due to close proximity of arm to the source. (Reproduced with permission from Vañó *et al* [8]).

**Figure 10:**
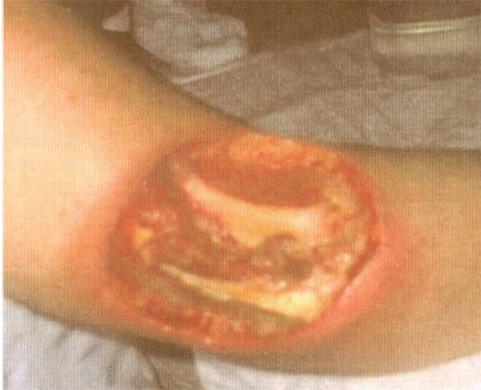
Injury to arm of patient. Patient was draped for procedure and physicians did not realize that she had moved her arm so that it was resting on the port of the X-ray tube during the procedure (Reproduced with permission from Wagner *et al* [19]).

**Figure 11:**
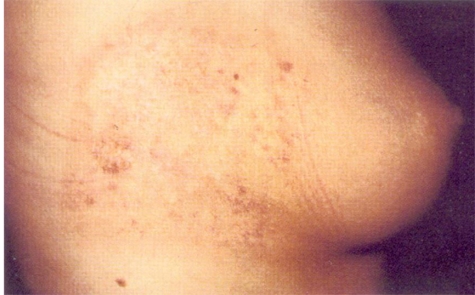
Injury to right flank in close proximity to right breast of 17-year-old girl after two procedures to treat her arrhythmia. (Reproduced with permission from Vañó *et al* [8]).

#### The position of the image receptor

With few exceptions, the image receptor should be placed as close to the patient as is practicable for the procedure. As the image receptor is moved closer to the patient, the output of the X-ray device decreases, thus decreasing dose rate to the patient.

#### Output settings of the equipment

The following are a few of the options or features available on many modern machines.

1. Variable pulsed fluoroscopy
2. Variable dose rate fluoroscopy
3. Variable dose level fluorography
4. Variable image rate fluorography
5. Collimator devices
6. Virtual collimation
7. Virtual patient positioning
8. Last image hold
9. Capture of last image hold
10. Last fluoroscopy replay

Physicians and technologists should be very familiar with such options and employ them as necessary. For example, most cardiology procedures can be performed at a fluoroscopic pulse rate of 15 pulses per second as opposed to a rate of 30 per second. The dose savings from this selection can be very considerable. A rate of 7.5 per second can be used for many vascular procedures. The physician should select the minimum rate that is consistent with the safe and efficient completion of the procedure. Similarly, many machines have dose rate selections that use different beam filters or different tube currents. The minimum dose rate consistent with the needs of the task should be employed. The same principles apply with respect to fluorographic frame rates and dose level settings. Settings should change with the progression of the procedure. Physicians should work with technologists on managing these settings. Technologists should assist the physician and be familiar with the physician’s procedure so that the technologist knows when different settings should be employed.

Physicists should be consulted on the settings. They can determine which settings actually save dose and by how much. For example, lower pulse rates for fluoroscopy do not always reduce the dose rate. Whether or not various settings actually reduce dose should be verified for every machine. The physicist can perform tests to assess the dose rates for each setting.

Last image hold is a very familiar feature on all modern machines. The last fluoroscopic frame is stored in memory and remains displayed on a video monitor once the X rays are turned off. A new feature on many units is fluoroscopy replay wherein the last 10-20 seconds of fluoroscopy is stored in memory. Replaying the fluoroscopy or using last image hold to study a procedure is a proven method of good dose management. Sometimes this image can be used to document the satisfactory placement of a device. Storing the image for this documentary purpose can save an additional radiation run in many cases.

The use of collimators to narrow the imaging field is also a recommended practice. Virtual collimator controls allow the physician to narrow the collimators without applying the X rays. The edges of the collimators are displayed by computer simulation using the last image hold for anatomic reference. Similarly, the table can be repositioned and the virtual positioning option uses last image hold to show the physician where the anatomy is being relocated in the image. No radiation is necessary.

Use of all the above tools and options in a wise and prudent manner will result in considerable dose savings to the patient with the added benefit of improved radiation limitation for personnel.

#### Dose monitoring

In all cases of radiation injury with which the author is familiar, the capability to monitor dose for the patient was either not used or not available.

At the author’s teaching hospital, a case of an unusually high radiation dose was investigated. The patient weighed 131 kg and was 1.7 m in height. The only dose monitor available was a kerma-area-product meter, which is known to be difficult to employ as a skin dose monitor [23, 24]. The patient had undergone a bi-plane electrophysiological and ablation procedure that involved 110 minutes of fluoroscopy with a dose-area product of 194,000 cGy * cm^2^. On the face of it, this could have resulted in a serious skin injury. The department had in place a policy that the radiation physicist would be called anytime the fluoroscopy time exceeded 40 minutes. The physicist could then make an assessment of the potential skin dose based on the kerma-area product. The policy also required the technologist to inform the physician of the prolonged procedure and that the physician should consider reorienting the beam so as to avoid irradiation of the same skin area. All these policies were followed for that particular procedure. The beam was re-oriented twice and the physicist was appropriately called to make sure policies were followed and to estimate the skin dose. This realistically saved the patient from harmful radiation dose buildup in the skin. The patient was visited by a nurse who examined the patient’s back six weeks after the procedure. No skin rashes or other indications of radiation injury were present.

This vignette demonstrates that sophisticated dosimetry equipment need not be available for a facility to establish sound policies on radiation management. All that needs to be in place is a procedure that permits the physician to make prudent judgments about radiation delivery during difficult procedures. While sophisticated dosimetry equipment is desired, lack of it does not preclude effective dose monitoring practices.

The use of fluoroscopy time as a surrogate measure for radiation dose is the least accurate method of determining risk to the patient [23, 24]. There are many reasons for this, the biggest being that it fails to record anything about serial imaging and provides no information relative to radiation output rates for different sizes of patients. But, as we have seen, it can be a valuable monitor for potential risk. While more informative than time, kerma-area product is likewise a poor method of dose assessment. It can be useful but usually requires assistance from a physicist or other experts in dosimetry.

**Figure 12:**
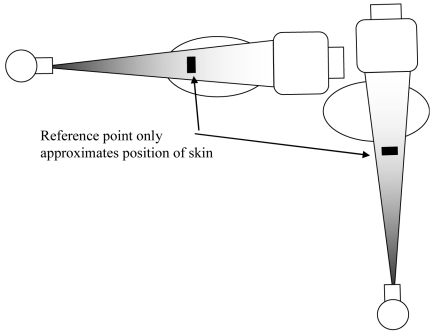
Dose reference point for lateral and PA beam orientations. Note that the reference measurement will be overestimated for PA orientation and underestimated in the lateral orientation due to the mismatch in position with the true skin position.

Another method of dose estimation is to monitor air kerma at a reference point. All modern machines have this capability. Usually, the air kerma at the reference point is cumulatively updated. For most angiographic equipment the reference point is located 15 cm from the isocentre and towards the X-ray source. This roughly approximates the position of the patient’s skin surface during cardiac procedures when the heart is positioned at the isocentre. It is more accurate than kerma-area-product, but has some deficiencies. These include the following:

1. the skin dose is roughly 40% greater than the indicated air kerma
2. the air kerma will be underestimated in some cases and overestimated in others (Figure12)
3. no accounting is made for risk to different skin sites when the beam is re-oriented

So, using air kerma at a reference point to estimate skin dose must be done with discretion. Some facilities use a 3, 6, 9 rule to help manage radiation delivery during difficult procedures. By this rule, the physician is advised when the reference air kerma reaches 3 Gy. This first alert is just for the physician’s information. The purpose is to help the physician gauge the pace of the procedure and to project just how much radiation might be necessary for its completion. The physician might wish to re-orient the beam. At 6 Gy, the second alert is provided. At this point, the physician knows that there is a risk of erythema or more severe effects if the beam has not been rotated to a new orientation. This gives the physician a chance to consider options for dose abatement. At 9 Gy, the third alert is issued. The degree of risk to the patient will depend on whether previous dose abatement actions have been implemented. This does represent a potentially serious dose level and a benefit-risk decision is necessary, just as a physician would make a benefit-risk decision about whether or not the iodine burden from the contrast agent is too great. Further warnings at 3 Gy intervals would be provided, with the physician making commensurate decisions about benefit versus risk.

**Figure 13:**
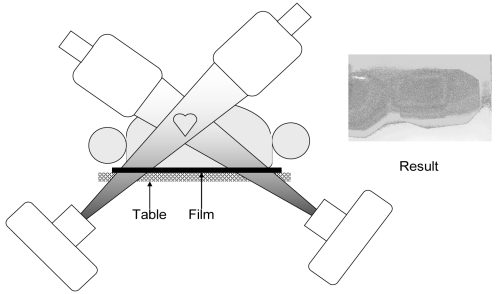
Special dosimetry “film” to monitor skin dose in patients (Specialty Products, Inc. Wayne, New Jersey, USA). The example shown is a biplane procedure. The film is placed flat on the table at the level where the beam will enter the patient. Note the different shapes of the fields, demonstrating changes in collimation and beam angle during the procedure. Note also the different darkness levels, indicating differences in skin dose with different locations. The field on the left was off the edge of the film, but it still provides useful data. (Reprinted with permission from: Wagner *et al* [19]).

Other methods for dose monitoring include computer dose-mapping programs and dosimetry film [24]. Computer dose-mapping programs are not easily acquired and the reader is referred to other articles on this method. A radiochromic dosimetry “film” (technically called media) [International Specialty Products, Incorporated, Wayne, New Jersey, USA] has special properties that permit it to be used to accurately assess skin dose (Figure 13). The film is not particularly sensitive to light. It is placed under the patient at the site where the beam enters the skin. As X rays pass through the film, the film turns black; no processing is required. The darkness of the film indicates the dose to the skin. To assess the dose, a calibration strip of film with different grey levels can be compared to the darkness on the procedure’s film. The method is easy to use and provides valuable dosimetry information [25]. During a prolonged procedure, if there is a concern over skin dose, the film can be removed and immediately examined for darkness to assess skin dose.

### After the procedure

Professional societies [20, 24] and others [26] recommend that patients should be advised about procedures that may have delivered high doses to the skin of a patient. They should be advised to report any skin changes. Specifically, the patient should be advised about the area on the skin of the back where a rash might develop. The patient should be asked to examine him- or herself about 2 to 3 weeks after the procedure for any skin changes in those areas. Some facilities place a follow-up call to the patient during this time to query about any skin irritation.

The benefits of these activities are as follows:

1. The patient knows ahead of time that this is a potential but rare event.
2. There is a mechanism for feedback on how often skin effects might be occurring. Data on erythema that eventually fades should create an action item to review the procedure. Information extracted from that review should be used to reassess procedures and improve them if necessary.
3. Should an erythema develop, the patient can be advised to see a dermatologist and the dermatologist should be contacted, advising him or her on the particular details of the patient’s complaint. For instance, you can advise the dermatologist where the rash would be located if it is a radiation-induced rash. Furthermore, the dermatologist knows to include radiation in the differential diagnoses.
4. If it is a radiation rash, the patient will have prompt knowledge about the cause and not be frustrated with incorrect diagnoses and unsatisfactory medical explanations about the progression of the lesion.

Without a follow-up, the patient leaves the facility with no knowledge about the potential skin effects. If an effect develops, the patient is not likely to associate it with the procedure, which was performed previously. If the patient seeks medical help for the rash, the physician might not realize that the angiographic procedure could cause the effect and will look for other diagnoses, all of which are incorrect. Care will be uncertain. And, the facility will have no feedback that this has occurred, leaving a false sense of security about the safety of future procedures.

## FINAL COMMENTS

This author recently received this e-mail:

“My husband was diagnosed with a biopsy in May 2006 with a radiation burn from several heart catherizations (sic). We have been seeing a wound specialist since June. Along with the wound, he has been suffering with severe burning and stabbing pain and trouble breathing. We have been to pulmonary specialists, thorasic (sic) surgeons, cardiologists and pain specialists all say they have no experience with a radiation burn. We are desperate for help in this matter…”

Only through education and adequate programs to monitor and manage radiation delivery during fluoroscopically-guided interventional procedures will we be able to stop this type of message from coming across our desk.
